# A Content Analysis of Video Advertisements for Dietary Supplements in Japan

**DOI:** 10.3390/healthcare9060742

**Published:** 2021-06-17

**Authors:** Reina Iye, Tsuyoshi Okuhara, Hiroko Okada, Rie Yokota, Takahiro Kiuchi

**Affiliations:** 1 Department of Health Communication, Graduate School of Medicine, The University of Tokyo, Tokyo 113-8655, Japan; yokotarie-tky@umin.ac.jp; 2 Department of Health Communication, School of Public Health, The University of Tokyo, Tokyo 113-8655, Japan; okuhara-ctr@umin.ac.jp (T.O.); okadahiroko-tky@umin.ac.jp (H.O.); tak-kiuchi@umin.ac.jp (T.K.)

**Keywords:** dietary supplements, advertising, health behavior, health communication, health promotion

## Abstract

Dietary supplements are widely advertised and the market is expanding worldwide. Research suggests that dietary supplement advertising may lead consumers to make inappropriate health-related decisions, to express behaviors such as overdosing, and to neglect healthy lifestyle behaviors. We conducted a content analysis of video advertisements for dietary supplements and described the content of advertisements with high numbers and frequent views. We analyzed 82 video advertisements on YouTube that promoted fat-reduction effects. We extracted 22 themes and classified them into 10 categories. The categories with the highest numbers of advertisements were “Exemption” (i.e., consuming the product frees the audience from refraining from binge eating) (20 ads, 24.4%) and “Health Concerns” (i.e., the product intake solves the health concerns of the audience) (19 ads, 23.2%). These advertisements may stimulate negative audience attitudes toward appropriate health behaviors. The category with the most frequent views was “Lifestyle” (i.e., adding product intake to a healthy lifestyle) (3,035,298 views). “Lifestyle” advertisements portray physical activity in a positive light and may promote appropriate health behaviors in the audience. We discuss the possible effects of the advertisements on audiences and consider issues for future research and practice.

## 1. Introduction

Health promotion is the process of enabling people to control and improve their own health and its determinants [1]. When choosing lifestyles and behaviors, individuals are sometimes influenced by the environment [1]. Both individual and environmental factors prompt the selection of inappropriate health behaviors. The development of an environment that promotes healthy lifestyles and diet is important from the perspective of health promotion.

Many adults take dietary supplements to maintain and improve their health [2,3]. The US Drug Supplement Health and Education Act defines a dietary supplement as “a product (other than tobacco) that is taken in addition to the normal diet. Furthermore, dietary supplements must contain one or more dietary ingredients, including vitamins, minerals, herbs or other botanicals, amino acids, and other substances such as enzymes, organ tissues, glandular materials, and metabolites” [4]. The National Institutes of Health provides this definition: “Dietary supplements come in a variety of forms, including tablets, capsules, gummies, and powders, as well as drinks and energy bars [5]”. On the basis of these statements, we defined dietary supplements in this study as various forms of foods that contain functional ingredients. There are a range of dietary supplements available in Japan, including tablets, beverages, snacks, and fresh foods. The Japanese government only permits pharmaceutical companies to label their products as affecting the structure or functioning of the body. Food products, including dietary supplements, are not permitted to make such claims. The exceptions are Foods for Specified Health Uses (FOSHU) (individual permission system), Foods with Nutrient Function Claims (FNFC) (prior notification system), and Foods with Function Claims (FFC) (self-certification system). The labels of these products can feature health claims according to the Japanese Food with Health Claims system [6].

FOSHU are food products for which specific health claims are made and contain ingredients that are expected to affect physiological functions [7]. The Japanese Consumer Affairs Agency (CAA) examines the effects and safety of each food and permits appropriate labeling. FFC differ from FOSHU in that they are not individually reviewed by the CAA [7]. Information on safety, functionality, and other aspects of FFC are reported to the Director General of the CAA before sales, and the business operator is responsible for labeling FFC according to their functions [7]. Both FOSHU and FFC are designed to be consumed by healthy people [7]. Businesses are allowed to label the nutrient functions of FNFC according to the standards set by the Japanese Ministry of Health, Labour and Welfare [7].

The market for dietary supplements is expected to expand worldwide [8]. In Japan, the market size for FOSHU increased in the 20 years following 1997 by approximately 500% [9]. The market for FFC increased by approximately 500% in the 5 years following 2015 [10]. Japanese consumers are mainly interested in dietary supplements that claim to have an effect on the reduction of fats such as neutral fat, visceral fat, and body fat [10,11]. FOSHU that claim to reduce neutral fat and body fat account for 24.4% of product sales, and the market for dietary supplements that claim to reduce blood neutral fat and body fat is also expected to expand [12,13].

However, several systematic reviews and meta-analyses have shown that most dietary supplements are ineffective in preventing or treating disease [14,15,16]. Furthermore, serious safety issues and drug–supplement interactions have been reported [17,18,19]. Nonetheless, dietary supplements are widely advertised. The main sources of information about dietary supplements are the Internet and video advertisements (ads) [3,20]. The expansion of the fifth-generation mobile communication system (5G) has also increased the placement of video ads [21]. Previous studies have shown that video ads for food products influence the eating behavior of audiences [22,23,24,25,26,27]. The health benefit claims of many dietary supplement ads are unfounded, and there is little mention of potential risks [28,29]. Advertising for dietary supplements is often misleading, and consumers may believe that dietary supplements prevent or treat disease [30,31]. Many consumers believe that the claims of dietary supplement ads are generally true [32,33]. Accordingly, advertising of dietary supplements may have negative effects on consumers, such as a reduction in appropriate lifestyles and overconsumption of dietary supplements with associated adverse events [27,34,35,36,37,38].

Even if dietary supplements are consumed, it is important to adopt appropriate lifestyle habits, such as a well-balanced diet and moderate physical activity and exercise [39]. The Ministry of Health, Labour and Welfare have advised the public that “A good way to incorporate dietary supplements is to use them to gradually improve your diet and lifestyle” [40]. In Japan, the content of dietary supplement ads is mainly regulated under the Health Promotion Act and the Act against Unjustifiable Premiums and Misleading Representations. These laws regulate the labeling of health maintenance and promotion products. For example, they do not permit claims that slimming effects can be obtained without concomitant lifestyle improvement, labels that make excessive claims, labels that are contrary to the facts, or advertising that substantially misleads people and hinders the improvement of national health [41]. Using the Act against Unjustifiable Premiums and Misleading Representations, the CAA can order businesses that have made unjustified representations to pay surcharges [41].

For example, in 2016, the CAA announced orders of action to 16 businesses selling dietary supplements containing kudzu flower-derived isoflavone as a functional ingredient. Nine of the businesses were also ordered to pay surcharges. The media advertisements (including video ads) of these nine businesses claimed that the intake of dietary supplements alone could easily produce a recognizable waistline slimming effect without any physical activity or dietary improvement [42]. In 2020, the CAA established a guideline stating that whether or not ads are misleading is judged from the impressions and perceptions that the general public receives from the label as a whole, not from specific words or pictures alone [43]. The US Federal Trade Commission has also issued the same caution regarding ads for dietary supplements [44].

However, research in the field of public health has paid little attention to regulating and improving the communication between businesses and consumers regarding the advertising of dietary supplements [45]. As mentioned above, video ads for dietary supplement products may influence consumers’ health-related perceptions and behaviors. Content analyses of social networking, magazines, radio, and websites have been conducted [30,46,47,48,49], but few studies have discussed the effects of dietary supplement video ads [50], and there has been no inductive analysis of video ads. Therefore, the present study examined the content of video ads for dietary supplements that promote a fat-reduction effect. The study aims were as follows: (1) to identify the themes and categories that describe dietary supplement video ads and the number of ads in each theme/category, (2) to identify the content characteristics of ads in those categories with high numbers of ads. We also discuss issues for future research and practice in light of the study findings.

## 2. Materials and Methods

### 2.1. Material Collection

**Figure 1:**
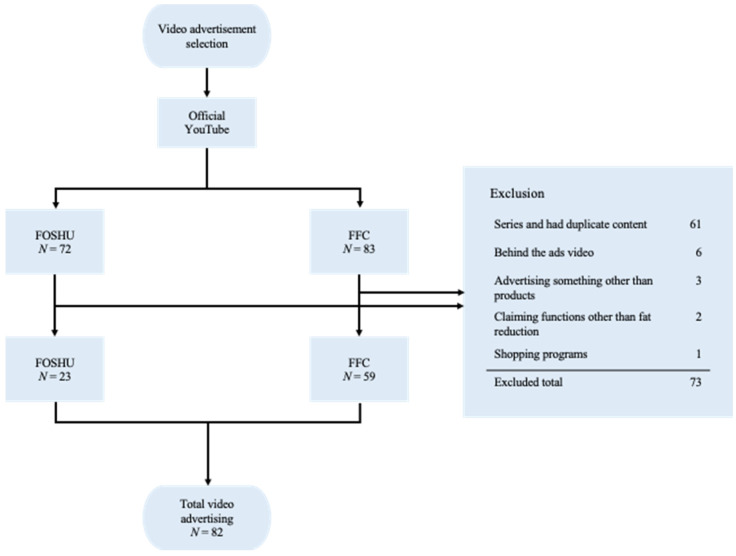
Flow diagram of advertisement selection.

The CAA publishes a list of FOSHU and FFC that are licensed or registered. This allowed us to systematically collect video ads. We analyzed video ads for FOSHU and FFC. The three main video sharing platforms in Japan are YouTube, Niko Niko Douga, and TikTok; their usage rates in 2019 were 76.4%, 17.4%, and 12.5%, respectively, clearly indicating that YouTube is the most popular platform [51]. Additionally, in Japan, many food companies and brands have their own YouTube advertising accounts [52]. Therefore, we used videos published on YouTube for the analysis. We used the list of FOSHU and FFC published on the CAA website [53,54] to identify products with functional labeling that claimed a fat-reduction effect. The first author (RI) checked the official YouTube website for relevant products and extracted ads related to the products identified. We excluded the following ads: ads that were produced as a series and had duplicate content; ads that advertised something other than products; ads that claimed functions other than fat reduction; behind the ads video; and shopping programs. RI extracted the videos on 5 October 2020. Figure 1 shows the flow of the extraction of ads.

### 2.2. Content Characteristics

RI coded the ads in terms of their content characteristics and extracted basic information about the ads, their main characters, and the lifestyle that they depicted. Table A1 shows the coding items. In ads featuring two or more characters, the character who appeared most and was most involved in the theme of the ad was considered to be the main character. Because we considered it important to capture the characteristics of ads from a public health perspective, the presence and ratio of time devoted to lifestyle habits, diet, and physical activity were also coded.

### 2.3. Themes and Categories

A number of symbolic elements constitute video content [55]. A shot, scene, or sequence is the compositional unit of a video, which is a collection of symbolic elements [55]. A shot is the smallest unit and is a continuous single frame. A scene is a collection of shots constructed in a continuous spatial–temporal or semantic relationship. A sequence is a collection of meanings composed of multiple scenes. There can be little variability in the interpretation of the content of shots, which are the smallest unit of video composition. Describing the content of shots enables coders to reduce biases and differences in evaluation when they extract themes [55]. Therefore, we defined one video ad as one sequence, and inductively extracted the theme of each video ad. Then we classified the extracted themes into categories. We used the following procedure.

The first author (RI) thoroughly watched the extracted videos and divided each video into shots. RI described the symbolic elements in each shot using Microsoft Excel, categorizing the elements as follows [55]. (1) Non-verbal images: for example, images with no verbal information; the behavior of characters; background images. (2) Verbal images: verbal information conveyed through images, such as message boards and charts. (3) Verbal audio: verbal information conveyed through audio, such as characters and narration. (4) Non-verbal audio: for example, background music and sound effects. Example shot descriptions are shown in Table A2, which was translated into English for this report.

RI integrated shots into a scene unit by judging the coherence of the meaning of the scenes. RI extracted notable descriptions (i.e., the core content that constitutes the meaning of each scene) from each scene and entered this into the Excel sheet (Table A2). By briefly summarizing the notable description, RI produced one rephrasing of the notable description for each scene. Then, RI generated a theme name to encompass the rephrasing of the notable description, and defined the theme. RI used this procedure for all videos. The theme names and their definitions were updated during the analysis. In addition, RI categorized themes and gave them category names, defining the superordinate concepts that encompassed the themes.

### 2.4. Interrater Reliability

To ensure interrater reliability of theme extraction and theme categorization, the fourth author (RY) independently evaluated the themes and categories. Using the theme categories and their definitions generated by the first author (RI), RY evaluated (1) which theme each video applied to, and (2) which category each theme applied to. RY learned the criteria for theme extraction and categorization for each theme during a 1-h meeting with RI. The author discussion was based on the coding manual. When a difference in the two authors’ evaluations arose, RI and RY discussed and selected the final theme and classification. RI described the final themes and categories, and the numbers and percentages of ads per theme and category. To assess interrater reliability, Cohen’s kappa was used to assess interrater agreement of the coding. Analyses were performed using IBM SPSS Statistics for Windows, Version 21.0 (IBM Corp., Armonk, NY, USA).

## 3. Results

### 3.1. Basic Characteristics

We identified 82 ads from 29 YouTube accounts. Of the 82 video ads, 62 advertised products from listed companies [56]. The total number of products was 38, and the total number of functional ingredients was 25. Table 1 shows the sample characteristics; 58.5% of the ads were published in 2020 and most were published in 2018–2020. The length of the ads ranged from 6 to 253 s, with 29 ads (35.4%) of 15 s, 13 ads (15.9%) of 6 s, 9 ads (11.0%) of 30 s, and 5 ads (6.1%) of 120 s. Ads with less than 1000 views/month accounted for 68.3% of the total number of ads, with a range of 4 views/month to 1.7 million views/month. A total of 41.5% of ads claimed effects on visceral fat, followed by effects on dietary fat, serum triglycerides, body fat, and subcutaneous fat. We found more ads for FFC than FOSHU (72.0% vs. 28.0%).

### 3.2. Themes, Categories, and Number of Ads

A total of 22 themes were extracted and categorized into 10 categories. Agreement between the coders on the extraction of themes was 63 out of 82 cases (76.8%), and the Cohen kappa coefficient was 0.748 (indicating substantial agreement). The coders completely agreed (100%) on the categorization of the 22 themes.

Regarding study aim (1), Table 2 shows the themes and categories, their definitions, the number and percentage of ads for each theme and category, the number of views and percentages, and medians for the number of views. The categories with the highest numbers of cases were “Exemption” (i.e., consuming the product frees the audience from refraining from binge eating) (20 ads, 24.4%) and “Health Concerns” (i.e., the product intake solves the health concerns of the audience) (19 ads, 23.2%). The category with the most frequent views was “Lifestyle” (i.e., adding product intake to a healthy lifestyle) (viewed 3,035,298 times, 54.0%), followed by “Health Concerns” (viewed 1,576,538 times, 28.1%).

The themes with the highest number of cases were “No need to resist the desire for binge eating if the product is consumed” (15 ads, 18.3%), followed by “Concerns about visceral fat and body shape” (9 ads, 11.0%). The theme with the most frequent views was “Physical activity has a positive impact on life” (viewed 2,990,555 times, 53.2%).

### 3.3. Content Characteristics of Categories with A Large Number of Cases and Views

Regarding study aim (2), Table 3 shows the content characteristics of the categories with more than 10 ads (Exemption and Health Concerns) and the category with the highest total number of views (Lifestyle). The Exemption, Health Concerns, and Lifestyle ads accounted for 56.1% of the 82 total ads analyzed and 87.6% of the total number of views.

The main character in the Exemption ads was in their 20 s in 10 ads (50.0%) and in their 50 s in 5 ads (42.1%). Descriptions of meals appeared in 15 (75.0%) of the Exemption ads. For ads containing a description of food, food appearance time ratio (Table A1 Coding item 15) was 0.42. None of the Exemption ads featured physical activity of 3 or more metabolic equivalent of tasks (METs). Of the Health Concerns ads, 14 (73.7%) featured men and 10 (52.6%) featured obese main characters. Two ads (10.5%) featured physical activity of 3 METs or more, but neither of these ads described the physical activity in a positive light. Of the Lifestyle ads, four (57.1%) featured physical activity of 3 METs or more, and all ads depicted physical activity in a positive light. The ratio of time showing physical activity of 3 METs or more to the length of the video was 0.55. All seven Lifestyle ads featured celebrity endorsers.

## 4. Discussion

We found that almost one in four ads (20 ads, 24.4%) were Exemption ads. The theme of “No need to resist the desire for binge eating if the product is consumed” was represented the most in the Exemption ads (15 ads, 18.3%). For example, one ad depicted a main character who wanted to binge eat, but resisted and instead consumed a dietary supplement. The character was then shown happily eating. One ad representing the theme “No need to resist the desire to eat if the product replaces other foods” depicted a main character’s desire to eat sweets (e.g., chocolate) and her resistance to this desire. The advertised chocolate product containing functional ingredients allowed the main character to eat freely. Both of the ads described above featured popular celebrities who have over a million Instagram followers, and both advertise popular products available at convenience stores and supermarkets in Japan. Thus, such ads were considered likely to influence Japanese consumers. Many of the Exemption ads were characterized by the appearance of food, and food appeared in non-verbal and visual images for more than one-third of the length of these ads. For example, the Exemption ad with the largest number of views (299,860 views/month) depicted a full-screen image of ramen noodles, accompanied by audio advertising promoting the deliciousness of the noodles. Responsiveness to visual cues (pictures and videos) is as strongly related to eating behavior and weight gain as responsiveness to real food [57]. Cue-induced urges affect choices about what, when, and how much to eat, gradually contributing to long-term caloric overconsumption and obesity [58]. Thus, the Exemption ads may stimulate the urge to eat and encourage eating behavior in audiences. Research indicates that emotions, especially guilt, can play a role in determining food consumption [59,60]. In the Exemption ads, the product was portrayed as justifying the desire to eat and making the consumer feel less guilty. Low-fat labeling affects perceptions of appropriate serving size, leading to overeating of snack foods [60]. This may be because low-fat labeling reduces the guilt of eating [60]. Similar to this phenomenon, audiences of the Exemption ads may justify their desire to eat and increase their food intake. The intake of FOSHU and FFC is considered an acceptable part of a balanced diet [40]. However, Exemption ads may mislead audiences by suggesting that if they consume the dietary supplements, they can eat as much as they want to (as shown in the ads) without simultaneously improving their diet.

There were 19 ads (23.2%) in the Health Concerns category (almost as many as in the Exemption category), but the number of views (1,576,538, 28.1%) was higher than the views of Exemption ads. The Health Concerns ads featured messages to evoke health concerns in the audience (e.g., “Are you concerned about your high serum triglycerides?”). The concerns depicted in these ads were related to test results such as clinical laboratory values and medical checkups. However, most dietary supplements are not effective in preventing or treating diseases [14,15,16]. Audiences may miss opportunities for treatment and lifestyle improvement by relying on dietary supplements to reduce their concerns about test results. In the Health Concerns ads, male (73.7% of the ads) and obese (52.6% of the ads) figures frequently appeared, some of who are popular celebrities in Japan who may have some influence on consumers. Emphasizing the similarity between the ad content and the audience characteristics influences the audience and changes their perceptions [61]. Accordingly, men who view the Health Concerns ads, especially those who are obese, may miss out on opportunities to engage in treatment and lifestyle changes.

Only 2 of the 39 Exemption and Health Concerns ads featured physical activity of three METs or more. Neither ad portrayed the physical activity in a positive light (e.g., a popular middle-aged Japanese actor training on a treadmill with a pained look). These ads may mislead people into believing that physical activity is tiresome and that health concerns can only be solved by intake of dietary supplements.

The Exemption and Health Concerns ads suggested that people can lose weight or improve their laboratory test results without improving their lifestyle behaviors; therefore, these ads may mislead audiences into believing that dietary supplement products are much more effective than they actually are [41]. Additionally, Easy Solutions ads (9 cases, 11.0%) may mislead audiences by suggesting that dietary supplements are easy solutions to health-related problems. Of the total number of ads, 58.5% were in the Exemption, Health Concerns, and Easy Solutions categories. According to the YouTube description box (text information displayed below each video), some of the ads in these categories were broadcast on television. Ads aired on television may influence a wider audience than ads posted only on YouTube. There are thus concerns about the undesirable effects on audiences of Exemption, Health Concerns, and Easy Solutions ads.

Lifestyle ads were the most viewed, with 3,035,298 views/month (54.0%). This may be related to the fact that these ads featured celebrities as main characters and advertised the products of large businesses. Four of the Lifestyle ads (57.1%) featured physical activity of 3 METs or more, and all portrayed physical activity in a positive light. For example, one ad depicted smiling celebrity characters who were actively walking to achieve good health. The positive response of celebrities to physical activity and healthy diet can serve as a model for audiences [62]. The content of the Lifestyle ads was based on the premise of appropriate physical activity and diet. Although the purpose of advertising is to promote product sales, an increase in Lifestyle ads would be useful in terms of health promotion, because such ads may a promote healthy lifestyle and have a positive effect on audiences.

### 4.1. Implications for Government and Public Health Institutions

Businesses should be controlled to reduce ads that may negatively affect audiences (such as those in the Exemption, Health Concerns, and Easy Solutions categories) and to increase Lifestyle ads, which may have a positive effect on audiences. Some public interest foundations hold workshops for businesses on appropriate food labeling, including advertising for dietary supplements [63,64]. Nevertheless, as this study shows, ads that may negatively affect audiences, such as those representing Exemption, Health Concerns, and Easy Solutions, have been placed. Whether or not an ad is misleading to audiences is not judged by specific words or images, but by the overall impression and resulting perceptions [43,44] This could explain why it is difficult to implement advertising regulations. Video advertising for dietary supplements could be a health promotion resource, considering the large number of views such ads attract. To enable businesses to create such ads on their own initiative, the government and public health institutions should not only regulate the content of dietary supplement advertising, but also collect and share with businesses good practice regarding ads that have a positive impact on audiences, such as Lifestyle ads.

### 4.2. Implications for Businesses

Businesses should actively participate in the aforementioned workshops and make improvements to their advertising. As mentioned above, the Ministry of Health, Labour and Welfare have advised the public that “A good way to incorporate dietary supplements is to use them to gradually improve your diet and lifestyle” [40]. Ideal ads would describe to audiences in specific terms “a good way” to use dietary supplements. Rather than circumventing the regulations, businesses should seek “a good way” to describe their products using ads that are unique to their companies or brands. This would facilitate the health improvements that are claimed for dietary supplements.

### 4.3. Limitations

This study has several limitations. As the study focused on ads released at a specific time, the themes extracted in this study may not be exhaustive or fully represent the themes of dietary supplement video ads in Japan that claim fat-reduction effects. It should also be noted that only ads published on YouTube were included in this study and the monthly viewing figures were not just for Japan. Therefore, the viewing frequency results may not be accurate or generalizable to Japanese consumers. However, we believe that the monthly views do reflect the influence of video ads in each theme/category. Although we made efforts to systematically extract and classify themes, author bias may have affected the coding results. Because we analyzed ads in Japanese, the study findings cannot be generalized to countries other than Japan. Finally, because we only analyzed the content of ads, how ads affect audience perception and behavior remains unknown and should be examined in future studies. The present findings should be interpreted cautiously in light of these limitations. However, to the best of our knowledge, this is the first study to analyze the content of video ads for dietary supplements and (as described above) the findings have important implications.

## 5. Conclusions

This study identified themes, categories, and frequencies of video ads for dietary supplements in Japan. Exemption and Health Concerns ads appeared particularly frequently. Exemption and Health Concerns ads suggest that people can improve their health without improving their lifestyle behaviors if they consume dietary supplements; these ads may encourage audiences to adopt a negative attitude toward engaging in appropriate health behaviors. In contrast, Lifestyle ads portrayed physical activity in a positive light and may encourage audiences to engage in appropriate health behaviors. Regulation of businesses and cooperation between businesses and public health institutions is needed to reduce the number of Exemption and Health Concerns ads (which may negatively affect audiences) and to increase the number of Lifestyle ads (which may have a positive impact on audiences).

## Figures and Tables

**Table 1:** Sample characteristics (*n* = 82).

Characteristics		*n* (%)
Release date	2020	48 (58.5)
	2019	21 (25.6)
	2018	9 (11.0)
	2017 and earlier	4 (4.9)
Length of the ad	6 s	13 (15.9)
	15 s	29 (35.4)
	30 s	9 (11.0)
	120 s	5 (6.1)
	Other	26 (31.7)
Number of views/month	0–999 times	56 (68.3)
	1000–1999 times	10 (12.2)
	≥2000 times	16 (19.5)
Meaning of “fat” in the labeling *	visceral fat	34 (41.5)
	dietary fat	28 (34.1)
	serum triglycerides	27 (32.9)
	body fat	19 (23.2)
	subcutaneous fat	14 (17.1)
Type of product	FOSHU	23 (28.0)
	FFC	59 (72.0)

* Some products claimed multiple functions, so the total number of cases does not add up to 82. The percentage indicates the ratio of ads to the number of samples (*n* = 82).

**Table 2:** Themes, categories, and their definitions.

Categories and Themes	Definition	*n* (%)	Number of Views/Month (%) *	Median Views/Month [Interquartile Range]
**Exemption**	Taking the product frees the audience from refraining from binge eating	20 (24.4)	308,408 (5.5)	276 [189–771]
No need to resist the desire for binge eating if the product is consumed	The product removes resistance to and concern about the intake of carbohydrates, fats, etc., and lets the audience freely eat whatever they want	15 (18.3)	307,188 (5.5)	
No need to resist the desire to eat if the product replaces other foods	If the audience replaces what they want to eat with the product, they don’t have to resist the desire to eat	5 (6.1)	1219 (0.0)	
**Health Concerns**	The product intake resolves the health concerns of the audience	19 (23.2)	1,576,538(28.1)	488 [135–48,559]
Concerns about visceral fat and body shape	Targeting concerns related to appearance, such as visceral fat (fat in the abdomen) and body shape	9 (11.0)	266,674 (4.7)	
Concerns about diet and medical test results	Targeting concerns about eating habits and clinical test values (e.g., triglycerides, blood sugar)	6 (7.3)	543,847 (9.7)	
Concerns about medical checkup results	Targeting concerns about medical checkups	4 (4.9)	766,018 (13.6)	
**Easy Solutions**	Suggesting the product is an easy solution to health-related issues	9 (11.0)	2039(0.0)	50 [33–101]
Issues about health can be easily solved with the product	Appealing to the ease of solving health-related issues, such as laboratory test values and waist size, using expressions such as “easy” and “simple”	7 (8.5)	435 (0.0)	
Multiple issues can be solved with one product	Claiming that multiple issues, such as sugar and fat intake, visceral fat, and subcutaneous fat, can be solved with a single product	2 (2.4)	1604 (0.0)	
**Lifestyle**	Adding product intake to healthy lifestyle	7 (8.5)	3,035,298 (54.0)	605 [454–625,608]
Increasing the attractiveness of engaging in physical activity	Recommending physical activity by increasing interest in sports or suggesting easy ways to engage in physical activity	4 (4.9)	44,138 (0.8)	
Physical activity has a positive impact on life	Describing physical activity in a positive way and suggesting that it will improve life	2 (2.4)	2,990,555 (53.2)	
Self-management of energy intake and integration of products into daily life can improve health	Suggesting self-management and integrating products into daily life by making audiences think about energy intake	1 (1.2)	605 (0.0)	
**Good Taste**	Product tastes good or goes well with meals	6 (7.3)	25,647 (0.5)	1363 [990–2739]
Product tastes good	Focusing on the taste and flavor of the product and highlighting its deliciousness	4 (4.9)	21,583 (0.4)	
Product’s taste goes well with meals	Focusing on the taste and flavor of the product to emphasize that it goes well with meals	2 (2.4)	4065 (0.1)	
**Explanations**	Explaining the product’s functionality or usage	5 (6.1)	912 (0.0)	171 [43–242]
Explanation of the product’s mechanism and functional ingredients	Explaining how functional ingredients work in the human body using graphs and diagrams	4 (4.9)	741 (0.0)	
Demonstration of the functionality and effectiveness of the product	Demonstrating the functionality and effectiveness of the product	1 (1.2)	171 (0.0)	
**Impressions**	Mostly non-verbal description, little verbal information	4 (4.9)	67 (0.0)	7 [5–19]
Description using non-verbal images	Using non-verbal images to show the happiness of those who consume the product	4 (4.9)	67 (0.0)	
**Direction to Online Information**	Directing audience to the product website or social networking sites	4 (4.9)	2091 (0.0)	416 [76–862]
Direct audience to the product website	Encouraging the audience to search by product name	3 (3.7)	1337 (0.0)	
Direct audience to social networking sites	Encouraging the audience to post product-related information on other social networking sites	1 (1.2)	754 (0.0)	
**Promotion of Approval**	Emphasizing that the product is approved by some public organizations	2 (2.4)	822 (0.0)	411 [281–541]
Emphasize that the product is FOSHU or FFC	Emphasizing that the product is FOSHU or FFC, which are approved by the government	2 (2.4)	822 (0.0)	
**Newness**	Emphasizing that the food or the ingredient is being reported as a dietary supplement for the first time	2 (2.4)	132,420 (2.4)	66,210 [33,632–98,789]
Newness of the product	Claiming that the product is the first food or functional ingredient to be reported as FOSHU or FFC	2 (2.4)	132,420 (2.4)	
**Others**	Ads not in the above categories	4 (4.9)	535,439 (9.5)	55,415 [558–188,717]
How to use product	Emphasizing that the product should be taken every day	1 (1.2)	702 (0.0)	
Recommendation to record products and body shape	Suggesting the use of an app or other method to record body shape	1 (1.2)	424,482 (7.6)	
Taking care of other people’s health	Necessity of taking care of the health of other people, such as family members	2 (2.4)	110,256 (2.0)	
		82 (100)	5,619,682 (100)	332 [104–1298]

* Sum of number of views/month in each theme or category. FOSHU, Foods for Specified Health Uses; FFC, Foods with Function Claims.

**Table 3:** Content characteristics of categories with a large number of cases and views.

Content Characteristics		Exemption (*n* = 20)	Health Concerns (*n* = 19)	Lifestyle (*n* = 7)	Total (*n* = 46)
Main character gender	Female, *n* (%)	13 (65.0)	5 (26.3)	2 (28.6)	20 (43.5)
	Male, *n* (%)	7 (35.0)	14 (73.7)	5 (71.4)	26 (56.5)
Main character age *	20 s, *n* (%)	10 (50.0)	3 (15.8)	3 (42.9)	16 (34.8)
	30 s, *n* (%)	2 (10.0)	5 (26.3)	0 (0.0)	7 (15.2)
	40 s, *n* (%)	2 (10.0)	2 (10.5)	3 (42.9)	7 (15.2)
	50 s, *n* (%)	5 (42.1)	8 (42.1)	1 (14.3)	14 (30.4)
	Over 60 s, *n* (%)	1 (5.26)	0 (0.0)	0 (0.0)	1 (2.2)
Type of endorser	Celebrity, *n* (%)	16 (80.0)	11 (57.9)	7 (100.0)	34 (73.9)
	Typical consumer, *n* (%)	4 (20.0)	7 (36.8)	0 (0.0)	11 (23.9)
	Anonymous spokesperson, *n* (%)	0 (0.0)	1 (5.3)	0 (0.0)	1 (2.2)
Body type of the main character *	Thin, *n* (%)	7 (35.0)	3 (15.8)	1 (14.3)	11 (23.9)
	Standard, *n* (%)	12 (60.0)	5 (26.3)	6 (85.7)	23 (50.0)
	Obese, *n* (%)	1 (5.0)	10 (52.6)	0 (0.0)	11 (23.9)
Number of ads in which food appears, *n* (%)		15 (75.0)	2 (10.5)	0 (0.0)	17 (37.0)
Food appearance time ratio (mean)		0.42	0.15	-	0.39
Number of ads featuring physical activity of ≥3 METs, *n* (%)		0 (0.0)	2 (10.5)	4 (57.1)	6 (13.0)
Physical activity of ≥3 METs time ratio (mean)		-	0.37	0.55	0.49
Number of ads in which physical activity of ≥3 METs was described positively, *n*		-	0	4	4

* One ad in Health Concerns was excluded because no person appeared in the image.

## Data Availability

Publicly available datasets were analyzed in this study. This data can be found here: https://www.youtube.com/ (accessed on 15 June 2021).

## Appendix A

**Table A1:** Coding items.

	Coding Items	Description
1	Ad title	Ad title on YouTube
2	Product name	The name of the product that the ad mainly shows
3	Name of functional ingredient (s)	The name of the ingredient(s) involved from the Consumer Affairs Agency database for the product(s) featured in the video ad
4	Length of the ad	Number of seconds the video lasts
5	Release date	Release date shown on YouTube
6	Number of views	Number of times the video ad was viewed on YouTube. The average number of views per month was calculated (rounded to one decimal place) to remove the effect of the release date
7	Meaning of “fat” in the labeling	“Approved labeling content” or “intended functionality” of the products appearing in the video ad from the Consumer Affairs Agency database. The first author inductively classified the meaning of “fat”
8	Type of product	Foods for Specified Health Uses (FOSHU) or Foods with Function Claims (FFC)
9	Business name	“Applicant’s name” or “Notifier’s name” of the product that mainly appears in the video ad from the Consumer Affairs Agency database
10	Body type of the main character (obese/standard/thin)	Body mass index of ≤18.5 was considered thin, BMI of ≥25 was considered obese, and other values were considered standard [65]
11	Main character gender	The first author guessed the main character’s gender as male or female
12	Main character age	The first author guessed the main character’s apparent age in 10-year increments (e.g., 20 s, 60 s)
13	Type of endorser [66]	The first author recorded the type of endorser of the main character, if the name or title of the performer was shown in the title or summary field of the video ad (e.g., celebrity, typical consumer, anonymous spokesperson)
14	Presence or absence of food	If food or dishes other than the product appeared in the video or non-verbal content, it was coded as (1)
15	Food appearance time ratio	Number of seconds in which food appeared as non-verbal images/number of seconds in video (e.g., if food or dishes appeared for 7 s out of a 10-s video, the ratio was 0.7)
16	Presence or absence of depiction of physical activity of ≥3 METs	If physical activity of ≥3 METs appeared as non-verbal and visual content, referring to the Physical Activity Standard 2013, it was coded as (1) [67,68]
17	Presence or absence of positive depiction of physical activity of ≥3 METs	If there was a non-verbal/visual description of physical activity of ≥3 METs caried out in a cheerful and joyful manner, it was coded as (1)
18	Physical activity of 3 METs appearance time ratio	Number of seconds in which physical activity of ≥3 METs appeared to number of seconds of non-verbal images in the video

**Table A2:** Examples of shot descriptions.

Time (Seconds)	Length of Shots	1. Non-Verbal Images	2. Verbal Images	3. Verbal Audio	4. Non-Verbal Audio	Notable Description	Rephrasing of the Notable Description
0	1	A man in his 20 s walks down a residential street while holding a product		It has become a habit to walk	Music for brass instrumentsSame as below	A man in his 20 s walks down a residential street while holding a product/It has become a habit to walk/Think I’ll go for 8000 steps/Swing arms wide	How nice it is to take a walk outside
1	1	A man in his 20 s looks around					
2	1	A man in his 20 s jumps while wearing sneakers		I think I’ll go for 8000 steps			
3	1	A man in his 20 s jumps					
4	0	A man in his 20 s walks while holding a product		[Product name] is my buddy			
4	1	A man in his 20 s walks down an alley in a residential area and turns		Explore a new route			
5	1	A cat walks down an alley in a residential area		Hmm?	Cat meowing		
6	2	A man in his 20 s walks on a bridge with his big strides		Swing arms wide			
8	1	A man in his 20 s peeks into a store		It’s so smart			
9	1	A man in his 20 s walks while carrying a product in a residential area					
10	1	A man in his 20 s smiles		Discovering the neighborhood … Just kidding			
11	1	A man in his 20 s drinks a product	Eat a balanced diet based on staple foods, main dishes, and side dishes. After drinking, recycle			A man in his 20 s drinks a product/I’ve started the [product name] life	Incorporate products into your life
12	2	A man in his 20 s and a man in his 50 s pose, pointing at a product while putting one foot up	I’ve started living on [product name]	I’ve started the [product name] life			
14	1	A man in his 20 s walks up the stairs smiling Product package FOSHU mark	Welcome, health!This product helps to reduce body fat through the action of quercetin glycoside, which activates lipolytic enzymes, and is therefore suitable for those who are concerned about body fat. The recommended dosage is 500 mL per day	Welcome, health!		Welcome, health!	Desire for good health
